# In-Utero Limb Amputation

**DOI:** 10.21699/jns.v6i1.399

**Published:** 2017-01-01

**Authors:** Vibha Sharma, KN Rattan, Nikhil Sharma

**Affiliations:** Department of Pediatric Surgery, PGIMS Rohtak, India

**Figure:**
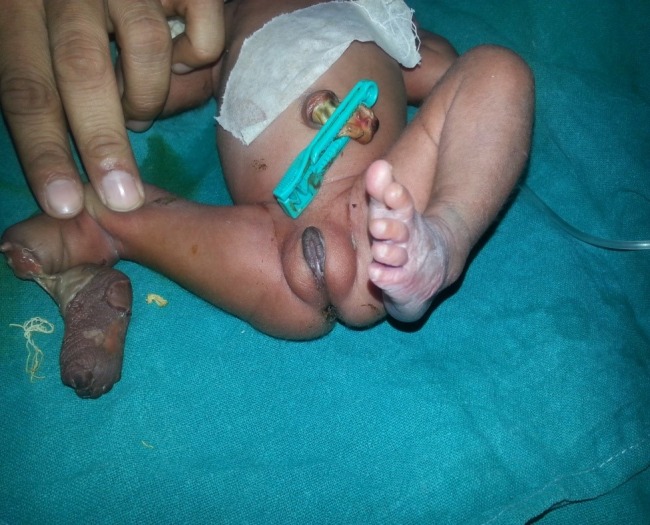
Figure 1: Devitalized amputated part of lower limb.

A preterm (32wks, 1.4 kg) female baby, born to a multigravida mother by emergency section in a tertiary care center (no history of consanguinity, exposure to teratogens, radiation, trauma, smoking or alcohol intake during pregnancy), presented with amputated right leg attached with a skin tag to the remaining part of right lower limb. No other defects were present. Ultrasound abdomen and cranium were normal (Fig.1).

Amniotic bands, also called constriction bands, congenital rings, Streeter dysplasia and annular defects are anomalous bands that encircle a digit or an extremity partially or completely; horizontally or spirally; superficially or deep. Severe constrictions can result in spontaneous intrauterine amputation.[1] Less severe bands may cause gross distal swelling and deformity, distal contractures, ulceration and syndactyly. Other anomalies include distal atrophy, limb-length discrepancy, anterolateral tibial bowing, hemihypertrophy, tibial pseudarthrosis and resistant teratologic clubfoot.[1] Upper extremity malformations are twice as common as lower extremity. But in our case malformation was at right lower limb. Non-limb anomalies include craniofacial clefts from bands about the head and face, cardiothoracic and internal organ defects from bands across the body, and renal abnormalities (37% of cases).[2] The bands can also cause skin scaring. Band formation have been associated with trauma, amniotic puncture, induced abortions, and chorionic villous sampling. In utero detection of amniotic bands is important for counseling and intra-uterine surgical repair. Amniotic bands be linear echoes floating in the amniotic fluid and connected to the fetal body on antenatal scan. Surgery depends on neurovascular status, distal swelling, and severity of the strictures. Minimal constrictions are simply released but severe constriction bands are released in multiple stages.[3] The most popular method of closure has been utilizing multiple Z-or W-plasties, other method are direct primary closure, saw-tooth approximation, and using triangular flaps.[4] Endoscopic in-utero release of amniotic bands has been successfully performed on human fetuses.

## Footnotes

**Source of Support:** Nil

**Conflict of Interest:** None
